# 2,2′-(9,9-Dioctyl-9*H*-fluorene-2,7-di­yl)bis­(4,4,5,5-tetra­methyl-1,3,2-dioxaborolane)

**DOI:** 10.1107/S1600536811049452

**Published:** 2011-11-25

**Authors:** Lin Huang

**Affiliations:** a Department of Chemistry and Biology, Xiangfan University, Xiangfan 441053, People’s Republic of China

## Abstract

In the title compound, C_41_H_64_B_2_O_4_, one of the five-membered rings has an envelope conformation, while the other, which may be affected by disorder, is nearly coplanar with the fluorene ring. The dihedral angle between the fluorene and dioxaborolane rings is 2.29 (1)°. Two of the methyl groups are disordered over two orientations in 0.67 (3):0.33 (3) and 0.568 (10):0.432 (10) ratios.

## Related literature

For the synthesis of the title compound, see: Pasini *et al.* (2003 ▶). For applications of the title compound, see: Cheon *et al.* (2005 ▶); Usta *et al.* (2006 ▶); Xie *et al.* (2006 ▶). For standard bond lengths, see: Allen *et al.* (1987 ▶).
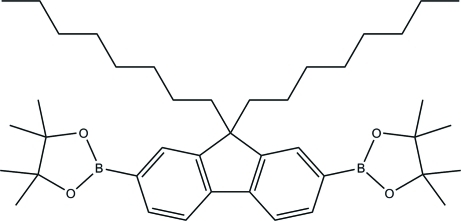


## Experimental

### 

#### Crystal data

C_41_H_64_B_2_O_4_
*M* *_r_* = 642.54
Triclinic, 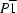

*a* = 12.8429 (16) Å
*b* = 13.4909 (17) Å
*c* = 14.1641 (18) Å
α = 110.369 (2)°
β = 90.183 (2)°
γ = 115.632 (2)°
*V* = 2039.6 (4) Å^3^
*Z* = 2
Mo *K*α radiation
μ = 0.06 mm^−1^
*T* = 296 K
0.16 × 0.12 × 0.10 mm

#### Data collection

Bruker SMART CCD area-detector diffractometer
Absorption correction: multi-scan (*SADABS*; Sheldrick, 1997 ▶) *T*_min_ = 0.917, *T*_max_ = 0.948
15861 measured reflections
7119 independent reflections
5313 reflections with *I* > 2σ(*I*)
*R*_int_ = 0.023

#### Refinement

*R*[*F* ^2^ > 2σ(*F* ^2^)] = 0.059
*wR*(*F* ^2^) = 0.189
*S* = 1.12
7119 reflections
476 parameters
44 restraints
H-atom parameters constrained
Δρ_max_ = 0.42 e Å^−3^
Δρ_min_ = −0.39 e Å^−3^

### 

Data collection: *SMART* (Bruker, 2001 ▶); cell refinement: *SAINT* (Bruker, 1999 ▶); data reduction: *SAINT*; program(s) used to solve structure: *SHELXS97* (Sheldrick, 2008 ▶); program(s) used to refine structure: *SHELXL97* (Sheldrick, 2008 ▶); molecular graphics: *SHELXTL* (Sheldrick, 2008 ▶); software used to prepare material for publication: *SHELXTL*.

## Supplementary Material

Crystal structure: contains datablock(s) I, global. DOI: 10.1107/S1600536811049452/ff2043sup1.cif

Structure factors: contains datablock(s) I. DOI: 10.1107/S1600536811049452/ff2043Isup2.hkl

Supplementary material file. DOI: 10.1107/S1600536811049452/ff2043Isup3.cml

Additional supplementary materials:  crystallographic information; 3D view; checkCIF report

## Figures and Tables

**Fig. 1.**
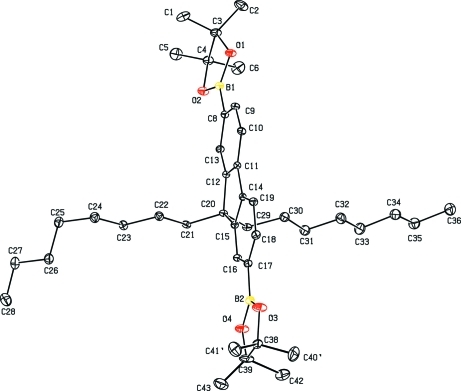
The title molecule with the atom-numbering scheme. The displacement ellipsoids are drawn at the 20% probability level and H atoms are omitted for clarity.

## supplementary crystallographic information

### Comment

Organoboron compounds have broad applications in organic synthesis, chemical sensing, and catalysis. For example the C—B bond in arylboronic acids and related derivatives is an extremely versatile synthon for constructing C—C bonds in catalyzed cross-coupling reactions with aromatic halides (commonly known as the Miyaura-Suzuki cross-coupling reaction). (Cheon *et al.*, 2005; Usta *et al.*, 2006; Xie *et al.*, 2006).

We herein report the crystal structure of the title compound (Fig. 1). One of the five membered rings (B1—O1—C3—C4—O2) has an envelope conformation, with maximum deviations from the plane of the fluorene ring of 0.374 (2), 0.113 (2), 0.202 (2) and 0.468 (2)Å for O1, C3, C4 and O2 respectively. The second five-membered ring (B2—O3—C38—C39—O4), which may be affected by disorder, is nearly co-planar with the fluorene ring. The dihedral angel between them is 2.29 (1) °. All bond lengths and angles are in normal ranges (Allen *et al.*, 1987). Two sets of methyl groups (C40/C40', C41/C41') and (C42/C42', C43/C43') are disordered over two orientations in a ratio of 0.67 (3):0.33 (3) and 0.57 (1):0.43 (1) respectively. The crystal is mainly stabilized by van der Waals forces.

### Experimental

The title compound (I) were prepared according to the reported method (Pasini *et al.*, 2003). Crystals of (I) suitable for X-ray data collection were obtained by slow evaporation of a CH_2_Cl_2_ and EtOH solution in a ratio of 5:2 at room temperature for one week.

### Refinement

Two sets of methyl groups (C40, C41) and (C42, C43) were found to be disordered over two orientations. The occupancies of the disordered positions (C40/C40', C41/C41') and (C42/C42', C43/C43') were refined to 0.67 (3):0.33 (3) and 0.57 (1):0.43 (1) respectively. All Hydrogen atoms were positioned geometrically (C—H = 0.93–0.97 Å) and refined as riding, allowing for free rotation of the methyl groups. The constraint *U*_iso_(H) = 1.2*U*_eq_(C) or 1.5*U*_eq_ (methyl C) was applied.

### Crystal data

**Table:**

C_41_H_64_B_2_O_4_	*Z* = 2
*M**_r_* = 642.54	*F*(000) = 704
Triclinic, *P*1	*D*_x_ = 1.046 Mg m^−^^3^
Hall symbol: -P 1	Mo *K*α radiation, λ = 0.71073 Å
*a* = 12.8429 (16) Å	Cell parameters from 5599 reflections
*b* = 13.4909 (17) Å	θ = 2.6–30.3°
*c* = 14.1641 (18) Å	µ = 0.06 mm^−^^1^
α = 110.369 (2)°	*T* = 296 K
β = 90.183 (2)°	Block, yellow
γ = 115.632 (2)°	0.16 × 0.12 × 0.10 mm
*V* = 2039.6 (4) Å^3^	

### Data collection

**Table:**

Bruker SMART CCD area-detector diffractometer	7119 independent reflections
Radiation source: fine-focus sealed tube	5313 reflections with *I* > 2σ(*I*)
graphite	*R*_int_ = 0.023
phi and ω scans	θ_max_ = 25.0°, θ_min_ = 1.6°
Absorption correction: multi-scan (*SADABS*; Sheldrick, 1997)	*h* = −15→15
*T*_min_ = 0.917, *T*_max_ = 0.948	*k* = −16→16
15861 measured reflections	*l* = −16→16

### Refinement

**Table:**

Refinement on *F*^2^	Primary atom site location: structure-invariant direct methods
Least-squares matrix: full	Secondary atom site location: difference Fourier map
*R*[*F*^2^ > 2σ(*F*^2^)] = 0.059	Hydrogen site location: inferred from neighbouring sites
*wR*(*F*^2^) = 0.189	H-atom parameters constrained
*S* = 1.12	*w* = 1/[σ^2^(*F*_o_^2^) + (0.1065*P*)^2^ + 0.2655*P*] where *P* = (*F*_o_^2^ + 2*F*_c_^2^)/3
7119 reflections	(Δ/σ)_max_ < 0.001
476 parameters	Δρ_max_ = 0.42 e Å^−^^3^
44 restraints	Δρ_min_ = −0.39 e Å^−^^3^

### Special details

**Table:**

Geometry. All e.s.d.'s (except the e.s.d. in the dihedral angle between two l.s. planes) are estimated using the full covariance matrix. The cell e.s.d.'s are taken into account individually in the estimation of e.s.d.'s in distances, angles and torsion angles; correlations between e.s.d.'s in cell parameters are only used when they are defined by crystal symmetry. An approximate (isotropic) treatment of cell e.s.d.'s is used for estimating e.s.d.'s involving l.s. planes.
Refinement. Refinement of *F*^2^ against ALL reflections. The weighted *R*-factor *wR* and goodness of fit *S* are based on *F*^2^, conventional *R*-factors *R* are based on *F*, with *F* set to zero for negative *F*^2^. The threshold expression of *F*^2^ > σ(*F*^2^) is used only for calculating *R*-factors(gt) *etc*. and is not relevant to the choice of reflections for refinement. *R*-factors based on *F*^2^ are statistically about twice as large as those based on *F*, and *R*- factors based on ALL data will be even larger.

### Fractional atomic coordinates and isotropic or equivalent isotropic displacement parameters (Å^2^)

**Table:**

	*x*	*y*	*z*	*U*_iso_*/*U*_eq_	Occ. (<1)
C1	1.3735 (2)	0.3651 (3)	0.3764 (3)	0.0806 (8)	
H1A	1.3487	0.3693	0.3145	0.121*	
H1B	1.4335	0.3400	0.3665	0.121*	
H1C	1.4041	0.4423	0.4311	0.121*	
C2	1.3038 (3)	0.2702 (3)	0.5025 (2)	0.0933 (10)	
H2A	1.3472	0.3498	0.5532	0.140*	
H2B	1.3519	0.2306	0.4918	0.140*	
H2C	1.2347	0.2271	0.5256	0.140*	
C3	1.2687 (2)	0.2753 (2)	0.40342 (18)	0.0563 (6)	
C4	1.2019 (2)	0.15304 (19)	0.31209 (18)	0.0541 (5)	
C5	1.2783 (3)	0.1184 (3)	0.2395 (2)	0.0851 (9)	
H5A	1.2299	0.0446	0.1833	0.128*	
H5B	1.3347	0.1089	0.2754	0.128*	
H5C	1.3186	0.1797	0.2140	0.128*	
C6	1.1277 (3)	0.0521 (3)	0.3440 (3)	0.0920 (10)	
H6A	1.0816	0.0760	0.3914	0.138*	
H6B	1.1778	0.0325	0.3763	0.138*	
H6C	1.0766	−0.0164	0.2848	0.138*	
B1	1.10573 (19)	0.26359 (19)	0.32660 (17)	0.0416 (5)	
C8	1.01169 (16)	0.29926 (17)	0.30368 (14)	0.0387 (4)	
C9	1.01592 (17)	0.40754 (17)	0.36800 (14)	0.0421 (5)	
H9	1.0777	0.4590	0.4232	0.051*	
C10	0.93147 (17)	0.43989 (16)	0.35192 (14)	0.0410 (4)	
H10	0.9364	0.5122	0.3955	0.049*	
C11	0.83889 (15)	0.36303 (15)	0.26974 (13)	0.0335 (4)	
C12	0.83306 (15)	0.25488 (15)	0.20272 (13)	0.0339 (4)	
C13	0.91887 (16)	0.22385 (16)	0.21998 (14)	0.0379 (4)	
H13	0.9148	0.1523	0.1756	0.045*	
C14	0.73640 (15)	0.37181 (16)	0.23552 (13)	0.0348 (4)	
C15	0.66891 (15)	0.26943 (15)	0.14792 (13)	0.0349 (4)	
C16	0.56717 (16)	0.25641 (17)	0.09998 (14)	0.0395 (4)	
H16	0.5223	0.1883	0.0420	0.047*	
C17	0.53113 (17)	0.34486 (18)	0.13794 (15)	0.0425 (5)	
C18	0.60018 (18)	0.44592 (18)	0.22592 (16)	0.0457 (5)	
H18	0.5768	0.5050	0.2520	0.055*	
C19	0.70171 (17)	0.46038 (17)	0.27493 (15)	0.0417 (4)	
H19	0.7462	0.5281	0.3333	0.050*	
C20	0.72435 (15)	0.18565 (15)	0.11903 (13)	0.0357 (4)	
C21	0.75758 (17)	0.16625 (17)	0.01160 (14)	0.0417 (4)	
H21A	0.8013	0.1211	0.0013	0.050*	
H21B	0.6856	0.1173	−0.0389	0.050*	
C22	0.82896 (19)	0.27659 (18)	−0.00990 (15)	0.0484 (5)	
H22A	0.8983	0.3298	0.0432	0.058*	
H22B	0.7827	0.3180	−0.0079	0.058*	
C23	0.8663 (2)	0.24673 (19)	−0.11365 (16)	0.0519 (5)	
H23A	0.9109	0.2038	−0.1153	0.062*	
H23B	0.7963	0.1934	−0.1660	0.062*	
C24	0.9392 (2)	0.3522 (2)	−0.14084 (19)	0.0623 (6)	
H24A	1.0037	0.4109	−0.0844	0.075*	
H24B	0.8910	0.3886	−0.1492	0.075*	
C25	0.9891 (2)	0.3205 (2)	−0.23854 (19)	0.0661 (7)	
H25A	1.0466	0.3938	−0.2430	0.079*	
H25B	1.0299	0.2762	−0.2329	0.079*	
C26	0.9001 (2)	0.2484 (2)	−0.33613 (19)	0.0630 (6)	
H26A	0.8665	0.2967	−0.3464	0.076*	
H26B	0.8372	0.1801	−0.3284	0.076*	
C27	0.9479 (3)	0.2039 (3)	−0.4314 (2)	0.0736 (7)	
H27A	1.0097	0.2722	−0.4402	0.088*	
H27B	0.9829	0.1568	−0.4208	0.088*	
C28	0.8573 (3)	0.1300 (4)	−0.5278 (2)	0.1048 (11)	
H28A	0.7946	0.0634	−0.5192	0.157*	
H28B	0.8926	0.1015	−0.5837	0.157*	
H28C	0.8268	0.1779	−0.5421	0.157*	
C29	0.64286 (17)	0.06309 (16)	0.12104 (15)	0.0434 (5)	
H29A	0.5724	0.0263	0.0702	0.052*	
H29B	0.6819	0.0135	0.1000	0.052*	
C30	0.6060 (2)	0.0617 (2)	0.22205 (18)	0.0579 (6)	
H30A	0.5611	0.1055	0.2415	0.069*	
H30B	0.6755	0.1015	0.2746	0.069*	
C31	0.5322 (2)	−0.0648 (2)	0.21612 (19)	0.0660 (7)	
H31A	0.4593	−0.1009	0.1685	0.079*	
H31B	0.5738	−0.1105	0.1883	0.079*	
C32	0.5034 (3)	−0.0744 (2)	0.3151 (2)	0.0753 (8)	
H32A	0.4544	−0.0361	0.3393	0.090*	
H32B	0.5757	−0.0311	0.3650	0.090*	
C33	0.4407 (3)	−0.2015 (2)	0.3100 (2)	0.0767 (8)	
H33A	0.3659	−0.2436	0.2637	0.092*	
H33B	0.4872	−0.2416	0.2819	0.092*	
C34	0.4194 (2)	−0.2087 (2)	0.4121 (2)	0.0715 (7)	
H34A	0.3710	−0.1703	0.4391	0.086*	
H34B	0.4941	−0.1640	0.4588	0.086*	
C35	0.3606 (3)	−0.3333 (2)	0.4101 (2)	0.0738 (7)	
H35A	0.2863	−0.3786	0.3626	0.089*	
H35B	0.4096	−0.3713	0.3847	0.089*	
C36	0.3384 (3)	−0.3377 (3)	0.5127 (2)	0.0866 (9)	
H36A	0.4112	−0.2912	0.5607	0.130*	
H36B	0.3048	−0.4189	0.5070	0.130*	
H36C	0.2850	−0.3058	0.5361	0.130*	
B2	0.4188 (2)	0.3322 (2)	0.08212 (19)	0.0488 (6)	
C38	0.2767 (2)	0.3777 (2)	0.0435 (2)	0.0611 (6)	
C39	0.2543 (2)	0.2548 (3)	−0.0344 (2)	0.0851 (9)	
C40	0.1778 (6)	0.3834 (9)	0.0988 (9)	0.093 (3)	0.67 (3)
H40A	0.1623	0.3371	0.1402	0.139*	0.67 (3)
H40B	0.1084	0.3520	0.0496	0.139*	0.67 (3)
H40C	0.1997	0.4648	0.1418	0.139*	0.67 (3)
C41	0.3233 (13)	0.4701 (11)	−0.0030 (10)	0.127 (4)	0.67 (3)
H41A	0.3515	0.5482	0.0499	0.191*	0.67 (3)
H41B	0.2615	0.4562	−0.0519	0.191*	0.67 (3)
H41C	0.3865	0.4647	−0.0367	0.191*	0.67 (3)
C42	0.1504 (5)	0.1564 (5)	−0.0060 (6)	0.103 (2)	0.568 (10)
H42A	0.1427	0.0790	−0.0461	0.154*	0.568 (10)
H42B	0.0782	0.1582	−0.0201	0.154*	0.568 (10)
H42C	0.1677	0.1729	0.0655	0.154*	0.568 (10)
C43	0.2241 (8)	0.2131 (9)	−0.1497 (3)	0.118 (3)	0.568 (10)
H43A	0.2893	0.2610	−0.1738	0.177*	0.568 (10)
H43B	0.1563	0.2208	−0.1668	0.177*	0.568 (10)
H43C	0.2076	0.1311	−0.1816	0.177*	0.568 (10)
C40'	0.1934 (17)	0.362 (2)	0.1209 (16)	0.141 (10)	0.33 (3)
H40D	0.1866	0.2978	0.1389	0.211*	0.33 (3)
H40E	0.1174	0.3454	0.0909	0.211*	0.33 (3)
H40F	0.2242	0.4347	0.1813	0.211*	0.33 (3)
C41'	0.2797 (18)	0.4763 (12)	0.0103 (13)	0.072 (4)	0.33 (3)
H41D	0.2870	0.5429	0.0695	0.108*	0.33 (3)
H41E	0.2084	0.4457	−0.0362	0.108*	0.33 (3)
H41F	0.3456	0.5017	−0.0231	0.108*	0.33 (3)
C42'	0.1352 (6)	0.1440 (7)	−0.0788 (11)	0.140 (5)	0.432 (10)
H42D	0.1411	0.0880	−0.1400	0.210*	0.432 (10)
H42E	0.0778	0.1662	−0.0948	0.210*	0.432 (10)
H42F	0.1119	0.1081	−0.0296	0.210*	0.432 (10)
C43'	0.2997 (9)	0.2856 (9)	−0.1295 (6)	0.102 (3)	0.432 (10)
H43D	0.3716	0.3590	−0.1061	0.153*	0.432 (10)
H43E	0.2416	0.2938	−0.1645	0.153*	0.432 (10)
H43F	0.3133	0.2225	−0.1755	0.153*	0.432 (10)
O1	1.18195 (12)	0.31982 (13)	0.41729 (11)	0.0534 (4)	
O2	1.12008 (13)	0.17226 (13)	0.25826 (11)	0.0565 (4)	
O3	0.37970 (16)	0.41406 (17)	0.11369 (14)	0.0825 (6)	
O4	0.35039 (15)	0.23804 (16)	−0.00401 (14)	0.0759 (5)	

### Atomic displacement parameters (Å^2^)

**Table:**

	*U*^11^	*U*^22^	*U*^33^	*U*^12^	*U*^13^	*U*^23^
C1	0.0436 (14)	0.0717 (17)	0.116 (2)	0.0235 (13)	0.0014 (14)	0.0286 (16)
C2	0.107 (2)	0.138 (3)	0.0654 (18)	0.092 (2)	−0.0010 (16)	0.0268 (17)
C3	0.0499 (13)	0.0632 (14)	0.0600 (14)	0.0372 (11)	−0.0022 (10)	0.0145 (11)
C4	0.0532 (13)	0.0539 (12)	0.0622 (14)	0.0331 (11)	0.0056 (10)	0.0195 (10)
C5	0.091 (2)	0.090 (2)	0.084 (2)	0.0639 (18)	0.0190 (16)	0.0163 (16)
C6	0.092 (2)	0.0718 (18)	0.128 (3)	0.0404 (17)	0.021 (2)	0.0540 (19)
B1	0.0374 (12)	0.0436 (12)	0.0424 (12)	0.0184 (10)	0.0037 (9)	0.0155 (10)
C8	0.0338 (10)	0.0447 (10)	0.0380 (10)	0.0168 (8)	0.0040 (8)	0.0181 (8)
C9	0.0368 (10)	0.0445 (10)	0.0344 (10)	0.0140 (8)	−0.0052 (8)	0.0099 (8)
C10	0.0433 (11)	0.0381 (10)	0.0355 (10)	0.0187 (9)	0.0009 (8)	0.0078 (8)
C11	0.0328 (9)	0.0358 (9)	0.0310 (9)	0.0147 (8)	0.0046 (7)	0.0136 (7)
C12	0.0320 (9)	0.0358 (9)	0.0309 (9)	0.0141 (8)	0.0020 (7)	0.0116 (7)
C13	0.0374 (10)	0.0373 (9)	0.0380 (10)	0.0188 (8)	0.0038 (8)	0.0116 (8)
C14	0.0349 (10)	0.0385 (9)	0.0340 (9)	0.0180 (8)	0.0074 (7)	0.0161 (8)
C15	0.0329 (9)	0.0374 (9)	0.0360 (10)	0.0164 (8)	0.0048 (7)	0.0155 (8)
C16	0.0339 (10)	0.0426 (10)	0.0380 (10)	0.0164 (8)	0.0000 (8)	0.0130 (8)
C17	0.0390 (10)	0.0513 (11)	0.0453 (11)	0.0253 (9)	0.0095 (9)	0.0221 (9)
C18	0.0466 (11)	0.0503 (11)	0.0500 (12)	0.0313 (10)	0.0127 (9)	0.0187 (9)
C19	0.0435 (11)	0.0400 (10)	0.0393 (10)	0.0219 (9)	0.0056 (8)	0.0098 (8)
C20	0.0341 (10)	0.0360 (9)	0.0338 (9)	0.0170 (8)	−0.0020 (7)	0.0091 (7)
C21	0.0447 (11)	0.0439 (10)	0.0316 (10)	0.0230 (9)	−0.0018 (8)	0.0061 (8)
C22	0.0527 (12)	0.0509 (11)	0.0431 (11)	0.0268 (10)	0.0098 (9)	0.0161 (9)
C23	0.0562 (13)	0.0553 (12)	0.0463 (12)	0.0272 (11)	0.0125 (10)	0.0202 (10)
C24	0.0646 (15)	0.0573 (13)	0.0606 (14)	0.0241 (12)	0.0139 (12)	0.0234 (11)
C25	0.0584 (15)	0.0766 (16)	0.0697 (16)	0.0271 (13)	0.0222 (12)	0.0407 (13)
C26	0.0633 (15)	0.0770 (16)	0.0669 (15)	0.0385 (13)	0.0224 (12)	0.0398 (13)
C27	0.0791 (18)	0.0840 (18)	0.0736 (17)	0.0409 (15)	0.0347 (15)	0.0437 (15)
C28	0.110 (3)	0.140 (3)	0.069 (2)	0.063 (2)	0.0244 (19)	0.040 (2)
C29	0.0393 (11)	0.0372 (10)	0.0450 (11)	0.0138 (8)	−0.0050 (8)	0.0115 (8)
C30	0.0544 (13)	0.0518 (12)	0.0603 (14)	0.0151 (11)	0.0057 (11)	0.0262 (11)
C31	0.0634 (15)	0.0588 (14)	0.0667 (16)	0.0162 (12)	0.0050 (12)	0.0300 (12)
C32	0.0764 (18)	0.0681 (16)	0.0673 (16)	0.0176 (14)	0.0081 (13)	0.0311 (13)
C33	0.0750 (18)	0.0675 (16)	0.0829 (19)	0.0230 (14)	0.0168 (15)	0.0367 (14)
C34	0.0692 (17)	0.0677 (15)	0.0754 (17)	0.0263 (13)	0.0072 (13)	0.0324 (13)
C35	0.0725 (17)	0.0656 (16)	0.0860 (19)	0.0290 (14)	0.0127 (14)	0.0359 (14)
C36	0.087 (2)	0.096 (2)	0.085 (2)	0.0403 (17)	0.0201 (16)	0.0460 (17)
B2	0.0443 (13)	0.0602 (14)	0.0517 (14)	0.0297 (12)	0.0098 (11)	0.0253 (12)
C38	0.0472 (13)	0.0878 (17)	0.0750 (16)	0.0436 (13)	0.0127 (11)	0.0449 (14)
C39	0.0682 (17)	0.108 (2)	0.0802 (18)	0.0588 (16)	−0.0179 (13)	0.0149 (15)
C40	0.075 (4)	0.129 (6)	0.094 (5)	0.064 (4)	0.032 (3)	0.043 (4)
C41	0.097 (7)	0.164 (8)	0.174 (8)	0.059 (6)	0.037 (6)	0.126 (7)
C42	0.080 (4)	0.091 (4)	0.114 (5)	0.032 (3)	−0.003 (3)	0.023 (3)
C43	0.110 (5)	0.144 (5)	0.096 (4)	0.073 (4)	−0.013 (3)	0.024 (3)
C40'	0.101 (11)	0.29 (3)	0.166 (17)	0.136 (16)	0.081 (12)	0.18 (2)
C41'	0.064 (8)	0.085 (7)	0.095 (8)	0.044 (6)	0.022 (6)	0.053 (6)
C42'	0.127 (6)	0.144 (6)	0.148 (7)	0.072 (5)	−0.012 (4)	0.043 (4)
C43'	0.111 (5)	0.110 (5)	0.087 (5)	0.069 (4)	−0.008 (3)	0.017 (3)
O1	0.0498 (8)	0.0613 (9)	0.0492 (9)	0.0370 (7)	−0.0065 (7)	0.0071 (7)
O2	0.0565 (9)	0.0565 (9)	0.0517 (9)	0.0333 (8)	−0.0054 (7)	0.0066 (7)
O3	0.0767 (12)	0.0906 (13)	0.0821 (13)	0.0640 (11)	−0.0161 (9)	0.0031 (10)
O4	0.0681 (11)	0.0818 (12)	0.0751 (12)	0.0503 (10)	−0.0183 (9)	0.0075 (9)

### Geometric parameters (Å, °)

**Table:**

C1—C3	1.530 (4)	C27—H27B	0.9700
C1—H1A	0.9600	C28—H28A	0.9600
C1—H1B	0.9600	C28—H28B	0.9600
C1—H1C	0.9600	C28—H28C	0.9600
C2—C3	1.505 (4)	C29—C30	1.512 (3)
C2—H2A	0.9600	C29—H29A	0.9700
C2—H2B	0.9600	C29—H29B	0.9700
C2—H2C	0.9600	C30—C31	1.522 (3)
C3—O1	1.462 (2)	C30—H30A	0.9700
C3—C4	1.554 (3)	C30—H30B	0.9700
C4—O2	1.456 (2)	C31—C32	1.485 (4)
C4—C6	1.509 (4)	C31—H31A	0.9700
C4—C5	1.519 (4)	C31—H31B	0.9700
C5—H5A	0.9600	C32—C33	1.520 (4)
C5—H5B	0.9600	C32—H32A	0.9700
C5—H5C	0.9600	C32—H32B	0.9700
C6—H6A	0.9600	C33—C34	1.501 (4)
C6—H6B	0.9600	C33—H33A	0.9700
C6—H6C	0.9600	C33—H33B	0.9700
B1—O1	1.362 (3)	C34—C35	1.505 (4)
B1—O2	1.362 (3)	C34—H34A	0.9700
B1—C8	1.551 (3)	C34—H34B	0.9700
C8—C13	1.401 (3)	C35—C36	1.498 (4)
C8—C9	1.404 (3)	C35—H35A	0.9700
C9—C10	1.377 (3)	C35—H35B	0.9700
C9—H9	0.9300	C36—H36A	0.9600
C10—C11	1.388 (3)	C36—H36B	0.9600
C10—H10	0.9300	C36—H36C	0.9600
C11—C12	1.406 (2)	B2—O3	1.345 (3)
C11—C14	1.467 (2)	B2—O4	1.350 (3)
C12—C13	1.385 (2)	C38—O3	1.438 (3)
C12—C20	1.522 (2)	C38—C41	1.509 (6)
C13—H13	0.9300	C38—C40	1.511 (5)
C14—C19	1.391 (3)	C38—C39	1.536 (4)
C14—C15	1.401 (2)	C38—C40'	1.537 (9)
C15—C16	1.383 (2)	C38—C41'	1.545 (8)
C15—C20	1.526 (2)	C39—O4	1.438 (3)
C16—C17	1.400 (3)	C39—C43	1.518 (4)
C16—H16	0.9300	C39—C42'	1.527 (4)
C17—C18	1.401 (3)	C39—C43'	1.585 (4)
C17—B2	1.556 (3)	C39—C42	1.596 (4)
C18—C19	1.378 (3)	C40—H40A	0.9600
C18—H18	0.9300	C40—H40B	0.9600
C19—H19	0.9300	C40—H40C	0.9600
C20—C29	1.538 (3)	C41—H41A	0.9600
C20—C21	1.546 (3)	C41—H41B	0.9600
C21—C22	1.512 (3)	C41—H41C	0.9600
C21—H21A	0.9700	C42—H42A	0.9600
C21—H21B	0.9700	C42—H42B	0.9600
C22—C23	1.521 (3)	C42—H42C	0.9600
C22—H22A	0.9700	C43—H43A	0.9600
C22—H22B	0.9700	C43—H43B	0.9600
C23—C24	1.510 (3)	C43—H43C	0.9600
C23—H23A	0.9700	C40'—H40D	0.9600
C23—H23B	0.9700	C40'—H40E	0.9600
C24—C25	1.530 (3)	C40'—H40F	0.9600
C24—H24A	0.9700	C41'—H41D	0.9600
C24—H24B	0.9700	C41'—H41E	0.9600
C25—C26	1.501 (4)	C41'—H41F	0.9600
C25—H25A	0.9700	C42'—H42D	0.9600
C25—H25B	0.9700	C42'—H42E	0.9600
C26—C27	1.522 (3)	C42'—H42F	0.9600
C26—H26A	0.9700	C43'—H43D	0.9600
C26—H26B	0.9700	C43'—H43E	0.9600
C27—C28	1.500 (4)	C43'—H43F	0.9600
C27—H27A	0.9700		
			
C3—C1—H1A	109.5	H28A—C28—H28B	109.5
C3—C1—H1B	109.5	C27—C28—H28C	109.5
H1A—C1—H1B	109.5	H28A—C28—H28C	109.5
C3—C1—H1C	109.5	H28B—C28—H28C	109.5
H1A—C1—H1C	109.5	C30—C29—C20	116.80 (16)
H1B—C1—H1C	109.5	C30—C29—H29A	108.1
C3—C2—H2A	109.5	C20—C29—H29A	108.1
C3—C2—H2B	109.5	C30—C29—H29B	108.1
H2A—C2—H2B	109.5	C20—C29—H29B	108.1
C3—C2—H2C	109.5	H29A—C29—H29B	107.3
H2A—C2—H2C	109.5	C29—C30—C31	111.86 (19)
H2B—C2—H2C	109.5	C29—C30—H30A	109.2
O1—C3—C2	108.58 (19)	C31—C30—H30A	109.2
O1—C3—C1	106.07 (19)	C29—C30—H30B	109.2
C2—C3—C1	110.8 (2)	C31—C30—H30B	109.2
O1—C3—C4	102.25 (16)	H30A—C30—H30B	107.9
C2—C3—C4	115.6 (2)	C32—C31—C30	115.2 (2)
C1—C3—C4	112.7 (2)	C32—C31—H31A	108.5
O2—C4—C6	106.18 (19)	C30—C31—H31A	108.5
O2—C4—C5	109.11 (19)	C32—C31—H31B	108.5
C6—C4—C5	110.3 (2)	C30—C31—H31B	108.5
O2—C4—C3	102.21 (15)	H31A—C31—H31B	107.5
C6—C4—C3	113.6 (2)	C31—C32—C33	114.5 (2)
C5—C4—C3	114.7 (2)	C31—C32—H32A	108.6
C4—C5—H5A	109.5	C33—C32—H32A	108.6
C4—C5—H5B	109.5	C31—C32—H32B	108.6
H5A—C5—H5B	109.5	C33—C32—H32B	108.6
C4—C5—H5C	109.5	H32A—C32—H32B	107.6
H5A—C5—H5C	109.5	C34—C33—C32	113.5 (2)
H5B—C5—H5C	109.5	C34—C33—H33A	108.9
C4—C6—H6A	109.5	C32—C33—H33A	108.9
C4—C6—H6B	109.5	C34—C33—H33B	108.9
H6A—C6—H6B	109.5	C32—C33—H33B	108.9
C4—C6—H6C	109.5	H33A—C33—H33B	107.7
H6A—C6—H6C	109.5	C33—C34—C35	114.8 (2)
H6B—C6—H6C	109.5	C33—C34—H34A	108.6
O1—B1—O2	113.09 (17)	C35—C34—H34A	108.6
O1—B1—C8	123.48 (18)	C33—C34—H34B	108.6
O2—B1—C8	123.43 (18)	C35—C34—H34B	108.6
C13—C8—C9	118.03 (17)	H34A—C34—H34B	107.5
C13—C8—B1	121.32 (17)	C36—C35—C34	113.5 (2)
C9—C8—B1	120.64 (17)	C36—C35—H35A	108.9
C10—C9—C8	122.08 (17)	C34—C35—H35A	108.9
C10—C9—H9	119.0	C36—C35—H35B	108.9
C8—C9—H9	119.0	C34—C35—H35B	108.9
C9—C10—C11	119.10 (17)	H35A—C35—H35B	107.7
C9—C10—H10	120.4	C35—C36—H36A	109.5
C11—C10—H10	120.4	C35—C36—H36B	109.5
C10—C11—C12	120.32 (16)	H36A—C36—H36B	109.5
C10—C11—C14	131.25 (16)	C35—C36—H36C	109.5
C12—C11—C14	108.43 (15)	H36A—C36—H36C	109.5
C13—C12—C11	119.79 (16)	H36B—C36—H36C	109.5
C13—C12—C20	129.21 (16)	O3—B2—O4	112.59 (19)
C11—C12—C20	110.98 (15)	O3—B2—C17	123.8 (2)
C12—C13—C8	120.66 (17)	O4—B2—C17	123.6 (2)
C12—C13—H13	119.7	O3—C38—C41	100.9 (6)
C8—C13—H13	119.7	O3—C38—C40	111.6 (6)
C19—C14—C15	120.54 (17)	C41—C38—C40	111.6 (5)
C19—C14—C11	131.07 (17)	O3—C38—C39	103.50 (17)
C15—C14—C11	108.39 (15)	C41—C38—C39	110.9 (7)
C16—C15—C14	119.77 (17)	C40—C38—C39	116.9 (4)
C16—C15—C20	129.12 (16)	O3—C38—C40'	95.2 (9)
C14—C15—C20	111.11 (15)	C41—C38—C40'	130.8 (8)
C15—C16—C17	120.61 (18)	C40—C38—C40'	21.6 (6)
C15—C16—H16	119.7	C39—C38—C40'	109.9 (10)
C17—C16—H16	119.7	O3—C38—C41'	114.2 (7)
C16—C17—C18	118.28 (17)	C41—C38—C41'	23.4 (4)
C16—C17—B2	120.65 (18)	C40—C38—C41'	88.4 (5)
C18—C17—B2	121.05 (18)	C39—C38—C41'	122.2 (7)
C19—C18—C17	121.97 (18)	C40'—C38—C41'	108.5 (7)
C19—C18—H18	119.0	O4—C39—C43	112.3 (3)
C17—C18—H18	119.0	O4—C39—C42'	116.9 (5)
C18—C19—C14	118.83 (18)	C43—C39—C42'	68.3 (5)
C18—C19—H19	120.6	O4—C39—C38	104.88 (19)
C14—C19—H19	120.6	C43—C39—C38	125.0 (4)
C12—C20—C15	101.09 (13)	C42'—C39—C38	126.3 (5)
C12—C20—C29	111.91 (14)	O4—C39—C43'	97.1 (4)
C15—C20—C29	112.12 (15)	C43—C39—C43'	36.8 (4)
C12—C20—C21	111.41 (14)	C42'—C39—C43'	105.0 (5)
C15—C20—C21	111.89 (14)	C38—C39—C43'	101.5 (4)
C29—C20—C21	108.36 (14)	O4—C39—C42	99.1 (3)
C22—C21—C20	116.96 (15)	C43—C39—C42	104.9 (4)
C22—C21—H21A	108.1	C42'—C39—C42	36.9 (4)
C20—C21—H21A	108.1	C38—C39—C42	107.5 (3)
C22—C21—H21B	108.1	C43'—C39—C42	141.6 (4)
C20—C21—H21B	108.1	C38—C40—H40A	109.5
H21A—C21—H21B	107.3	C38—C40—H40B	109.5
C21—C22—C23	112.03 (17)	C38—C40—H40C	109.5
C21—C22—H22A	109.2	C38—C41—H41A	109.5
C23—C22—H22A	109.2	C38—C41—H41B	109.5
C21—C22—H22B	109.2	C38—C41—H41C	109.5
C23—C22—H22B	109.2	C39—C42—H42A	109.5
H22A—C22—H22B	107.9	C39—C42—H42B	109.5
C24—C23—C22	115.48 (18)	C39—C42—H42C	109.5
C24—C23—H23A	108.4	C39—C43—H43A	109.5
C22—C23—H23A	108.4	C39—C43—H43B	109.5
C24—C23—H23B	108.4	C39—C43—H43C	109.5
C22—C23—H23B	108.4	C38—C40'—H40D	109.5
H23A—C23—H23B	107.5	C38—C40'—H40E	109.5
C23—C24—C25	113.9 (2)	H40D—C40'—H40E	109.5
C23—C24—H24A	108.8	C38—C40'—H40F	109.5
C25—C24—H24A	108.8	H40D—C40'—H40F	109.5
C23—C24—H24B	108.8	H40E—C40'—H40F	109.5
C25—C24—H24B	108.8	C38—C41'—H41D	109.5
H24A—C24—H24B	107.7	C38—C41'—H41E	109.5
C26—C25—C24	115.2 (2)	H41D—C41'—H41E	109.5
C26—C25—H25A	108.5	C38—C41'—H41F	109.5
C24—C25—H25A	108.5	H41D—C41'—H41F	109.5
C26—C25—H25B	108.5	H41E—C41'—H41F	109.5
C24—C25—H25B	108.5	C39—C42'—H42D	109.5
H25A—C25—H25B	107.5	C39—C42'—H42E	109.5
C25—C26—C27	114.9 (2)	H42D—C42'—H42E	109.5
C25—C26—H26A	108.6	C39—C42'—H42F	109.5
C27—C26—H26A	108.6	H42D—C42'—H42F	109.5
C25—C26—H26B	108.6	H42E—C42'—H42F	109.5
C27—C26—H26B	108.6	C39—C43'—H43D	109.5
H26A—C26—H26B	107.5	C39—C43'—H43E	109.5
C28—C27—C26	114.1 (2)	H43D—C43'—H43E	109.5
C28—C27—H27A	108.7	C39—C43'—H43F	109.5
C26—C27—H27A	108.7	H43D—C43'—H43F	109.5
C28—C27—H27B	108.7	H43E—C43'—H43F	109.5
C26—C27—H27B	108.7	B1—O1—C3	107.17 (16)
H27A—C27—H27B	107.6	B1—O2—C4	107.55 (16)
C27—C28—H28A	109.5	B2—O3—C38	109.98 (19)
C27—C28—H28B	109.5	B2—O4—C39	108.92 (19)
